# How to Achieve Near-Normal Visual Acuity with Bevacizumab in Diabetic Macular Edema Patients

**DOI:** 10.3390/jcm10163572

**Published:** 2021-08-13

**Authors:** Bogumiła Sędziak-Marcinek, Adam Wylęgała, Elżbieta Chełmecka, Edward Wylęgała, Sławomir Teper

**Affiliations:** 1 Chair and Department of Ophthalmology, Faculty of Medical Sciences in Zabrze, Medical University of Silesia, Panewnicka 65 Street, 40-760 Katowice, Poland; wylegala@gmail.com (E.W.); slawomir.teper@gmail.com (S.T.); 2 Health Promotion and Obesity Management Unit, Department of Pathophysiology, Faculty of Medical Sciences in Katowice, Medical University of Silesia, Medyków 65, 40-728 Katowice, Poland; awylegala@sum.edu.pl; 3 Department of Statistics, Department of Instrumental Analysis, Faculty of Pharmaceutical Sciences, Medical University of Silesia, Ostrogórska 30 Street, 41-200 Sosnowiec, Poland; echelmecka@sum.edu.pl

**Keywords:** best-corrected visual acuity (BCVA), bevacizumab (IVB), diabetic macular edema (DME), diabetic retinopathy (DR), ultra-wide-field fluorescein angiography (UWFFA)

## Abstract

Patients suffering from diabetic retinopathy (DR) and diabetic macular edema (DME) are inherently interested in achieving normal or near-normal visual acuity. The study aimed to investigate factors influencing the visual acuity achieved by DME patients after bevacizumab (IVB) treatment. 98 patients (98 eyes) diagnosed with DR and DME underwent IVB treatment (9 injections/12 months). Patients were diagnosed and monitored using swept-source optical coherence tomography (SS-OCT), ultra-wide-field fluorescein angiography (UWFFA) and Early Treatment Diabetic Retinopathy Study (ETDRS) chart testing. We assessed macular central subfield thickness (CST), non-proliferative diabetic retinopathy (NPDR) indicators and best-corrected visual acuity (BCVA). After the treatment, patients were divided into BCVA_≤75_ and BCVA_>75_ groups. The IVB therapy increased the number of ETDRS letters read by about 9 and 8 in the BCVA_≤75_ and the BCVA_>75_ group, respectively. Before and after treatment, the BCVA_>75_ group had lower CST than the BCVA_≤75_ group. The treatment reduced macular CST by 177 μm in the BCVA_≤75_ group and only by 93 μm in the BCVA_>75_ group. Total non-perfusion area (NPA) decreased in both BCVA score groups after IVB therapy. Normal or near-normal vision can be achieved with IVB treatment, provided it starts when visual acuity is not significantly reduced yet. The ophthalmic screening of DR patients should also target those with relatively high visual acuity.

## 1. Introduction

Unmet patients’ expectations are a common cause of non-adherence and non-persistence in anti-VEGF therapy. Non-persistence is more often observed in patients suffering from diabetic macular edema (DME) than in patients with age-related macular degeneration (AMD). For this reason, achieving a treatment outcome that satisfies the patient is even more valuable as it may also increase the chances of the patient’s future compliance. Therefore, explaining and identifying the factors contributing to such an outcome should be a part of the pre-treatment evaluation [1].

Fluorescein angiography is an integral part of diabetic retinopathy (DR) diagnostics. It helps to identify microaneurysms (MA), non-perfusion areas (NPA), diabetic macular edema (DME) and neovascularization. Since much of the abnormalities in DR, especially NPA, can occur in the mid-periphery and periphery of the retina [2], ultra-wide-field fluorescein angiography (UWFFA) imaging is particularly useful in DR evaluation. UWFFA captures a wide field of the retina at once, allowing for visualization of many different retina areas at the same time point during angiography and reducing the scope of patient cooperation and the technical expertise in photography required from a specialist performing the examination. UWFFA also visualizes the periphery of the retina that previously was not photographed. UWFFA imaging proved that a substantial amount of DR abnormalities could develop in the peripheral area of the retina. Therefore, UWFFA imaging with Optos Advance system (Optos, Dunfermline, Scotland, UK), capturing twice as much of the retinal area as conventional digital acquisition systems [3], presents significant advantages over the conventional, recommended by the ETDRS [4], stereoscopic 7-field images (7R) in evaluating DR lesions [5,6,7].

Diabetic macular edema (DME) has been the leading cause of vision loss in diabetic patients [8]. Since diabetes in adults is estimated to affect 10.4% of the global population in 2040, the improvement of DME treatment becomes increasingly important [9]. Our previous study showed that the strict regimen of bevacizumab (IVB) intravitreal injections—9 injections over 12 months—decreased DME and DR severity in patients with and without retinal non-perfusion [10]. The study also showed that UWFFA allowed determining a satisfactory prognosis for patients with retinal non-perfusion and confirmed that patients with DME could be successfully treated with bevacizumab regardless of their non-perfusion status [10]. Nevertheless, the major factors helping achieve normal visual acuity in patients with DME after IVB treatment were not profoundly studied.

Diabetic patients suffering from DR and DME are inherently interested in achieving normal or near-normal visual acuity, defined as best-corrected visual acuity, BCVA, of more than 75 ETDRS letters [11,12,13]. Studies show that lower BCVA is associated with damage to the macula. Thus, we hypothesized that patients with higher BCVA, and less extensive damage to the macula, would benefit more from IVB treatment. The presented study aimed to identify factors that predict success or failure of bevacizumab treatment and investigated the factors influencing the final BCVA score, measured with ETDRS letters, achieved by patients with DME after IVB treatment in two subgroups of patients: showing unsatisfactory (BCVA_≤75_ group) and satisfactory (BCVA_>75_ group) response to the treatment. While some of the factors appear intuitive, such as baseline visual acuity, others still need to be explored. In most developed countries, diabetic patients are subjected to ophthalmological screening. Determining the most important factors that enable maintaining satisfactory visual acuity would improve the appropriate targeting of diabetic patients and the screening process.

## 2. Materials and Methods

Here we present the prospective observational study of 98 eyes of 98 consecutive patients diagnosed with diabetic retinopathy and diabetic macular edema (DME), who underwent bevacizumab treatment and were monitored using ultra-wide-field fluorescein angiography (UWFFA). The study presented here is an extension of the work by Sędziak-Marcinek et al. [10], where we described in detail both the diagnostic methodology and the general characteristics of patients enrolled in the study.

### 2.1. Permissions and Ethical Statements

The study followed the guidelines of the Declaration of Helsinki and was approved by the Ethics Committee of the Medical University of Silesia (decision KNW/0022/KB1/125/I/18/19). The study purpose, its protocol, as well as the benefits and possible risks related to the study were presented to the participants, who returned the written consent while enrolled in the study.

### 2.2. Study Design

The patients’ recruitment, diagnostics and intravitreal treatment were carried out in the outpatient clinic of the Clinical Department of Ophthalmology, Faculty of Medical Science, Medical University of Silesia throughout 2018–2020. The including and excluding criteria applied in the study were described in detail in the previous work by Sędziak-Marcinek et al. [10].

#### 2.2.1. Initial Diagnostics

The initial interview and examination were conducted during the routine appointment in the outpatient clinic. The initial interview aimed to gather the general medical and ophthalmological data. The initial examination with a slit lamp aimed to assess the anterior and posterior segments of the eye.

#### 2.2.2. Further Diagnostics

Further diagnostics aimed to assess the progress of diabetic macular edema, the severity of diabetic retinopathy and the level of visual acuity. This stage of diagnostics involved:

1. Swept-source optical coherence tomography (SS-OCT) that enabled the diagnose of diabetic macular edema based on central subfield thickness (CST) measurement and retinal morphology.
2. Ultra-wide-field fluorescein angiography (UWFFA) that enabled the evaluation of the non-perfusion areas (NPA), contrast leakage areas, microaneurysms (MA) count, diabetic retinopathy severity and differentiate between focal and diffuse macular edema according to SS-OCT data.
3. Early Treatment Diabetic Retinopathy Study (ETDRS) chart testing that enabled to assess best-corrected visual acuity (BCVA).

#### 2.2.3. Study Groups and Bevacizumab Treatment Protocol

Based on the including and excluding criteria and two-step diagnostics, 98 patients with diabetic macular edema were enrolled in the study, and 98 eyes were qualified for bevacizumab treatment.

The intravitreal injections were performed by an ophthalmologist. The eyes were locally anesthetized with proxymetacaine hydrochloride and disinfected with 5% iodine povidone. Then they were injected with 0.5 mg/0.05 mL bevacizumab (Avastin, Roche, Basel, Switzerland). Each eye received 9 injections over 12 months to reach the loading dose of bevacizumab [14,15,16,17]. The injections I-V were administered at monthly intervals, the injections VI-IX were administered bimonthly.

Each injection was preceded by macular central subfield thickness (CST) measurement with SS-OCT. In addition, before the first and one month after the last bevacizumab injection, the participants were subjected to the UWFFA and BCVA test to assess the effectiveness of the treatment.

Upon completing the bevacizumab treatment and initial analysis of the results, the patients were divided into two groups:

(1) BCVA_≤75_ group—patients who scored ≤ 75 ETDRS letters 1 month after completing the treatment (*n* = 41),
(2) BCVA_>75_ group—patients who scored > 75 ETDRS letters 1 month after completing the treatment (*n* = 57).

The detailed study design and flow of the patients during the experiment is presented in Table 1.

### 2.3. Diagnostic Methodology

#### 2.3.1. Swept-Source Optical Coherence Tomography (SS-OCT)

Swept-source optical coherence tomography (DRI OCT Triton tomograph, Topcon, Japan) used a 6 × 6 mm scanning protocol of a central macular field. The thickness at the fovea of the retina, here described as the central subfield thickness CST, was read against the ETDRS grid and expressed in μm. CST of 250 μm or bigger (≥250 μm) was determined as diabetic macular edema.

#### 2.3.2. Ultra-Wide-Field Fluorescein Angiography (UWFFA)

Ultra-wide-field fluorescein angiography (UWFFA) images were taken with the scanning laser ophthalmoscope Optos California P200DTx (Optos, Dunfermline, Scotland, UK) and processed using the OptosAdvance Software v4.2.31 that allows for accurate measurements of the visible retinal area in square millimeters (mm^2^) and adjusts for peripheral distortion. The preparation and image analysis were carried out according to the methodology by Fan et al. [5] and Fang et al. [17] exactly as described in the previous work [10]. The assessment of diabetic retinopathy parameters (non-perfusion areas, contrast leakage areas and microaneurysms count) and diabetic retinopathy severity were also carried out as described in the previous work [10].

#### 2.3.3. Best-Corrected Visual Acuity (BCVA) Assessment

##### Early Treatment Diabetic Retinopathy Study (ETDRS) Chart Testing

Best-corrected visual acuity was assessed using the ETDRS Charts (Precision Vision, La Salle, IL, USA). The ETDRS Chart R (manifest refraction), Charts 1 (right eye) and 2 (left eye) were printed with high-contrast lettering on a translucent white polystyrene panel. Chart was placed 4 m from the patient in a back-illuminated stand, lit from behind and displayed in a standard lightbox. The lightbox was illuminated by two fluorescent lamps with a reusable fenestrated sleeve (diffuser) that produced a chart luminance of 168 cd/m^2^, as recommended by the ETDRS protocol (80 to 320 cd/m^2^). The room lights were turned off during the visual acuity testing and the room illumination with the lights off was 2.0 f.c., as measured with LX 1010 BS Sinometer (Shenzhen, China). The refraction was checked with KR1-W autorefractometer (Topcon, Tokyo, Japan) and adjusted for manifest refraction using Chart R and placing sphere lenses and Jackson cross cylinders. The patient’s visual acuity was examined only for the studied eye with the final sphere and final cylinder. The vision testing started with the first letter on the top row of the chart for the examined eye (Chart 1 or 2). If a patient could not read more than 20 letters at a 4 m distance, the chart was moved 1 m to the patient. The testing proceeded according to the forced-choice paradigm from the top of the chart to the bottom. Patients were allowed to read the chart only one time, required to identify each letter and encouraged to guess if not sure. The examiner pointed to each new line. The patients’ responses were marked on a scoring sheet with correctly identified letters circled on the sheet. The ETDRS chart was scored using a single letter scoring method with credit given for any letter correctly identified. The testing was repeated with Chart 2 using the same rules and scoring procedures. 

### 2.4. Data Processing

#### 2.4.1. Bevacizumab Treatment Effectiveness Analysis

The relative macular central subfield thickness (CST_relative_) (Equation (1)) and relative best-corrected visual acuity (BCVA_relative_) (Equation (2)) were calculated to assess the effects of the therapy.

(1) $${\mathrm{CST}}_{\mathrm{relative}}=\frac{{\mathrm{CST}}_{\mathrm{after}\;\mathrm{therapy}}-{\mathrm{CST}}_{\mathrm{before}\;\mathrm{therapy}}}{{\mathrm{CST}}_{\mathrm{before}\;\mathrm{therapy}}}*100\%$$

(2) $${\mathrm{BCVA}}_{\mathrm{relative}}=\frac{{\mathrm{BCVA}}_{\mathrm{after}\;\mathrm{therapy}}-{\;\mathrm{BCVA}}_{\mathrm{before}\;\mathrm{therapy}}}{{\mathrm{BCVA}}_{\mathrm{before}\;\mathrm{therapy}}}*100\%$$

#### 2.4.2. Statistical Analysis

Qualitative variables are presented using percentages. The χ^2^ test was used to determine the relationship between non-measurable features, and Cohen’s kappa coefficient (*κ*) with 95% confidence intervals (CI) was calculated in case of a statistically significant relationship.

Quantitative variables are presented as mean and standard deviation (for data with normal distribution) or median and lower-upper quartiles (for data with non-normal or skewed distribution). Data distribution normality for quantitative characteristics at the beginning and the end of the therapy was checked with the Shapiro-Wilk test and quantile plots. Then the Student’s t-test for dependent samples or the non-parametric Wilcoxon pairwise test was used accordingly. The homogeneity of variances was checked with Levene’s test. The variables with non-normal distribution were analyzed using Friedman’s ANOVA test. In the central subfield thickness (CST) analysis, depending on the injection sequence number, an analysis of variance for repeated measurements with contrast analysis with Mauchly’s sphericity test was used. Whenever necessary, the normality of variables was improved using a logarithmic transformation. A linear regression model with the Pearson’s correlation coefficient (r) or Spearman’s rank correlation coefficient (ρ) were calculated to determine the correlation between quantitative variables. All statistical calculations were carried out using Statistica v. 13.3 program (TIBCO, Palo Alto, CA, USA), and statistical significance was set at *p* < 0.05.

## 3. Results

The study group consisted of 57 women and 41 men. In this case, 48 (49%) patients had a pseudophakic eye, and slightly more than half of them had a phakic eye (*n* = 50, 51%). Here, 48 (49%) eyes were diagnosed with focal and 50 (51%) eyes with diffuse macular edema. Half of the participants (*n* = 49) were diagnosed with retinal non-prefusion. Vascular leakage in the far periphery zone was noted in 34 (35%) eyes, in the mid-periphery zone in 37 (38%) eyes and in the posterior zone in 53 (54%) eyes. More than half of the participants were diagnosed with hypertension (*n* = 65, 66%) and hypercholesterolemia (*n* = 55, 56%). Here, 17 patients (17%) were affected by ischemic heart disease and seven (7%) by renal failure. In this case, 56 patients (57%) were subjected to insulin treatment, and 52 participants (53%) were undergoing anticoagulant treatment. The detailed characteristics of the study groups are presented in Table 2.

The analysis of the data collected during the initial examination and interview showed that patients of the BCVA_≤75_ group did not differ from the patients of the BCVA_>75_ group when it comes to general medical and ophthalmological variables (Table 2). The only difference was found for the severity of non-proliferative diabetic retinopathy that patients presented before the bevacizumab treatment started. Patients from the BCVA_≤75_ group were less often diagnosed with mild NPDR (15% vs. 35% for the BVCA_>75_ group), but more often with moderate NPDR (66% vs. 40% for the BCVA_>75_ group). 

**Figure 1:**
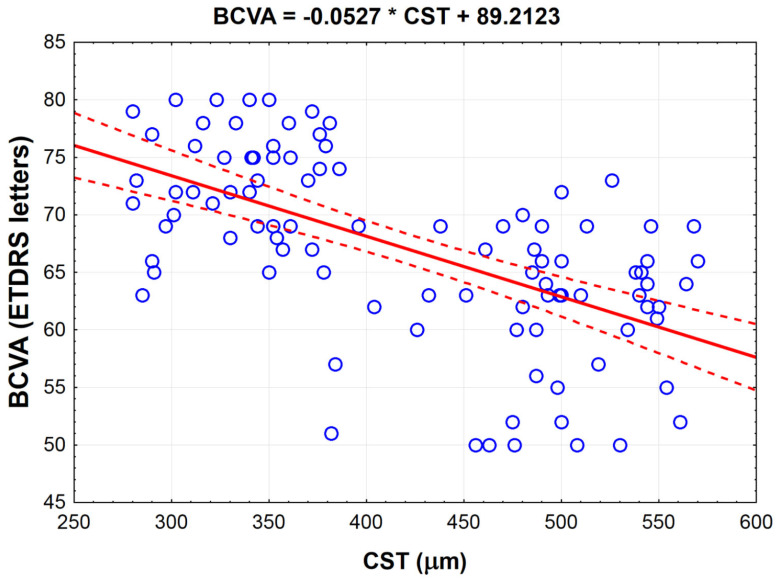
Linear regression between best-corrected visual acuity (BCVA) and central subfield thickness (CST) of patients (*n* = 98) with diabetic macular edema (DME) before bevacizumab therapy. Dashed lines denote 95% confidence interval.

The analysis of the variables measured for patients enrolled for this study showed that the best-corrected visual acuity (BCVA) negatively correlated with macular central subfield thickness (CST) (strong correlation, r = −0.591, *p* < 0.001) (Table 3). The negative linear correlation between BCVA and CST is described by the equation shown on Figure 1. Our analysis showed that for each 100 μm increase in CST, we can expect a decrease in patients’ BCVA by about 5.3 ETDRS letters. In addition, we found that BCVA negatively correlated with other parameters that are associated with diabetic retinopathy: total microaneurysms (MA) count (weak correlation, r = −0.285, *p* < 0.01), MA count in posterior zone (average correlation, r = −0.377, *p* < 0.001), total contrast leakage area (average correlation, ρ= −0.343, *p* < 0.001), contrast leakage area in far periphery area (weak correlation, ρ = −0.252, *p* < 0.05), mid-periphery area (average correlation, ρ = −0.315, *p* < 0.01) and in the posterior area (weak correlation, ρ = −0.287, *p* < 0.01). It also negatively correlated with total NPA (weak correlation, ρ = −0.272, *p* < 0.01), NPA in far periphery (average correlation (ρ = −0.303, *p* < 0.01). We found no correlation between BCVA and NPA in the posterior retinal zone (*p* = 0.309). BCVA did not relate to patients’ age (*p* = 0.602), diabetes duration (*p* = 0.818), BMI (*p* = 0.156) and glycated hemoglobin concentration in the serum (*p* = 0.142).

Similarly, macular CST did not correlate with patients’ age (*p* = 0.141) and diabetes duration (*p* = 0.647), BMI (*p* = 0.147). However, we found that CST positively correlated with glycated hemoglobin concentration in their serum (r = 0.268, *p* < 0.01, Table 2). We observed that CST was positively correlated with total MA count average correlation, r = 0.389, *p* < 0.001) and MA count in the posterior zone (strong correlation, r = 0.625, *p* < 0.001). It also positively correlated with total contrast leakage area (average correlation, ρ = 0.414, *p* < 0.001) and contrast leakage area in the posterior retinal zone (high correlation, ρ = 0.515, *p* < 0.001), and total NPA (weak correlation, ρ = 0.210, *p* < 0.05) and NPA in far periphery (weak correlation, ρ = 0.249, *p* < 0.05). We found no correlation between CST and contrast leakage area in the far (*p* = 0.104) and in the posterior zone (*p* = 0.748).

**Figure 2:**
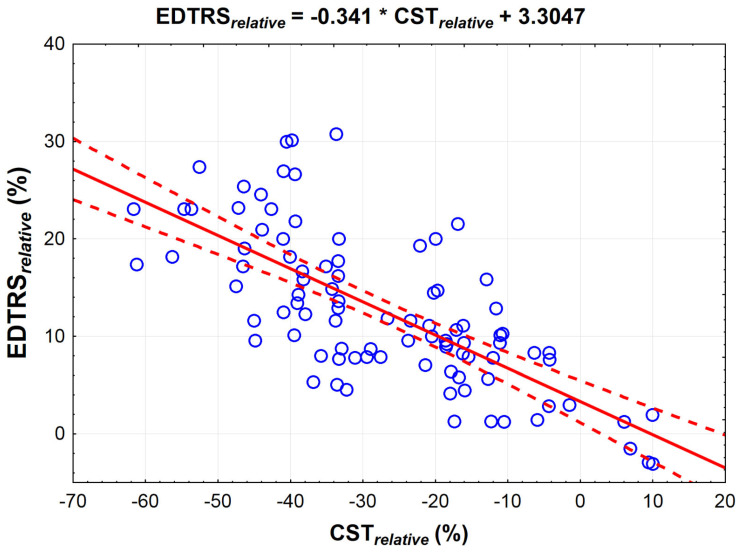
Linear regression between relative best-corrected visual acuity (BCVA_relative_) and relative central subfield thickness (CST_relative_) in patients with diabetic macular edema (DME) enrolled in the study (*n* = 98). Dashed lines denote 95% confidence interval.

We observed a high negative correlation (r = −0.7188) between BCVA_relative_ and CST_relative_ (*p* < 0.001) We found that a 10% increase in CST corresponds to a 3.4% decrease in the number of ETDRS letters read (Figure 2).

Non-proliferative diabetic retinopathy severity depended on the bevacizumab treatment (*p* < 0.001). Before the therapy, more than half of the patients showed moderate symptoms (51%), while after the therapy, the highest percentage (64%) of patients showed mild NPDR symptoms (Table 4).

The calculated concordance ratio indicated very weak compatibility of the results before and after the treatment (κ_Cohen_ = 2.28, 95% CI; 0.1–0.4) and suggested that the treatment changed the conditions of NPDR. In this case, 47 patients (48%) had no change in NPDR severity. We observed no NPDR severity deterioration case, but we saw improvement in NPDR severity: by one unit in 46 patients (47%) and two units in 5 patients (5%).

Non-proliferative diabetic retinopathy positively correlated with contrast leakage area and NPA in all retinal zones, and the strength of the correlation depended on the NPDR severity (Table 5).

**Figure 3:**
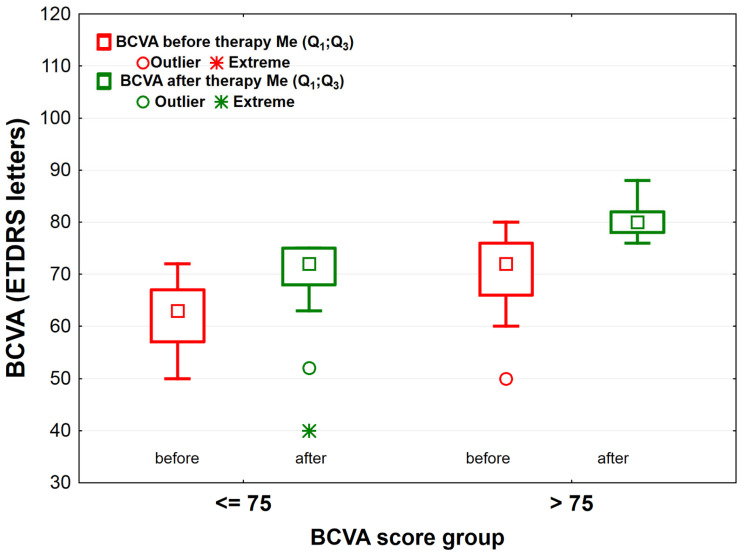
BCVA (best-corrected visual acuity) scores before and after bevacizumab treatment in the BCVA_≤75_ group (*n* = 41) and the BCVA_>75_ group (*n* = 57) of patients with diabetic macular edema (DME). Legend: squares—mean the median; rectangles—range 25–75%; whiskers indicate the range of non-outliers; circles indicate outliers; asterisks (*)—extreme values.

**Figure 4:**
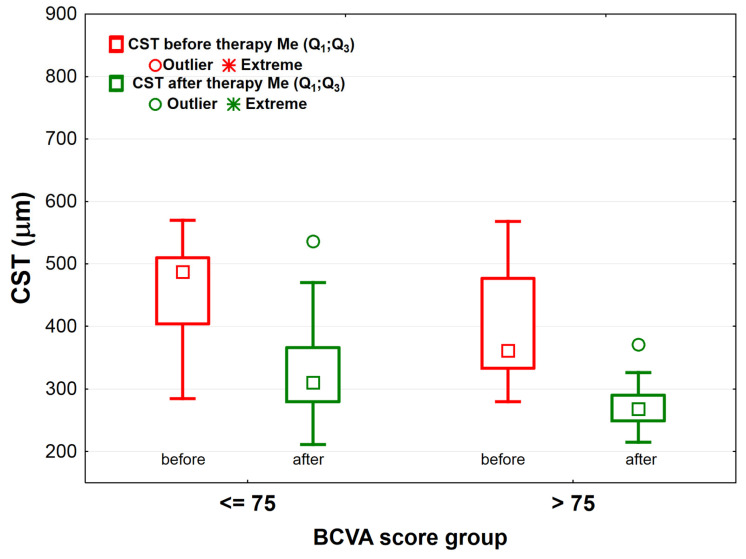
The macular CST (central subfield thickness) before and after bevacizumab treatment in the BCVA_≤75_ group (*n* = 41) and the BCVA_>75_ group (*n* = 57) of patients with diabetic macular edema (DME). Legend: squares—mean the median; rectangles—range 25–75%; whiskers indicate the range of non-outliers; circles indicate outliers; asterisks (*)—extreme values.

The bevacizumab therapy increased the number of letters read by the patients in both study groups: by about 9 ETDRS letters in the BCVA_≤75_ group—from 63.0 (57.0;67.0) to 72.0 (68.0;75.0) (*p* < 0.001) and by 8 ETDRS letters in the BCVA_>75_ group—from 72.0 (66.0;76.0) to 80.0 (78.0;82.0) (*p* < 0.001). Before and after the treatment, the BCVA_≤75_ group red less ETDRS letters when compared to the BCVA_>75_ group (*p* < 0.001 for both comparisons) (Figure 3). Similarly, significant differences in the macular CST were noted for both BCVA score groups before and after therapy. Before and after treatment the BCVA_>75_ group had lower CST than the BCVA_≤75_ group (difference before was 126 μm, *p* < 0.001; difference after was 42 μm, *p* < 0.001). In the BCVA_≤75_ group, the macular CST after the therapy was reduced by 177 μm on average—from 487.0 (404.0;510.0) μm to 310.0 (280.0;366.0) μm (*p* < 0.001), while in the BCVA_>75_ group only by about 93 μm—from 361.0 (333.0;477.5) μm to 268.0 (249.0;290.0) μm (*p* < 0.001) (Figure 4).

We observed that total contrast leakage area, contrast leakage area in the far periphery and posterior zone after bevacizumab therapy was reduced in each BCVA score group (*p* < 0.001 for both groups) (Table 6). Before the therapy, we found no statistically significant differences in total leakage area (*p* = 0.757), leakage area in the far periphery (*p* = 0.743) and in the posterior zone (*p* = 0.572) between the BCVA score groups. However, after therapy, the total contrast leakage area, contrast leakage area in the far periphery and the posterior zone were significantly higher in the BCVA_≤75_ group than in the BCVA_>75_ group (*p* < 0.05 for all variables) (Table 6). The analysis of the results showed that total NPA, NPA in the far periphery, in the mid-periphery and the posterior zone decreased in both BCVA score groups after bevacizumab therapy. We found no statistically significant differences between the BCVA_≤75_ and BCVA_>75_ groups in NPA occurrence before and after bevacizumab treatment.

**Figure 5:**
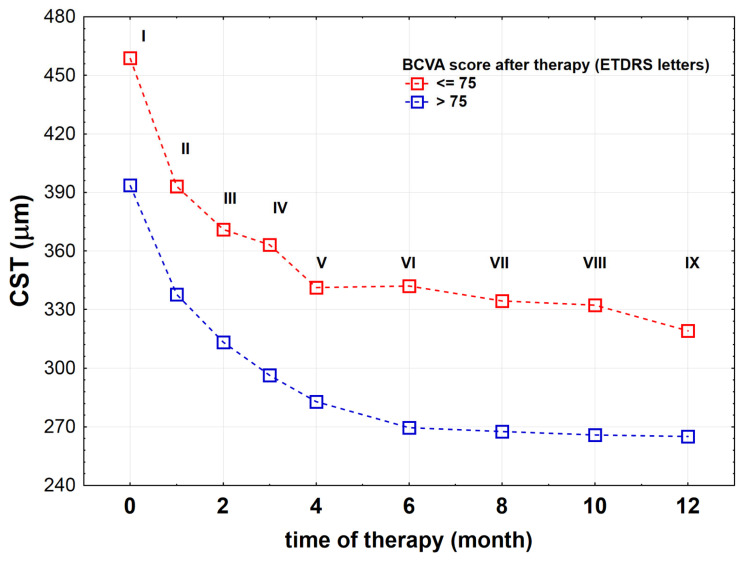
The macular CST (central subfield thickness) in the BCVA_≤75_ group (*n* = 41) and the BCVA_>75_ group (*n* = 57) of patients with diabetic macular edema (DME) during subsequent intravitreal bevacizumab injections.

We also checked how CST decreased during subsequent injections in both BCVA score groups. The comparative analyzes showed that the macular CST in the BCVA_>75_ group was lower than the macular CST in the BCVA_≤75_ score group after each subsequent injection (*p* < 0.001 for all time points) (Figure 5). Our analysis showed that the effect of intravitreal bevacizumab injections does not depend on gender. There were no statistically significant differences in the BCV_Arelative_ and CST_relative_ depending on gender (*p* = 0.494 and *p* = 0.923, respectively). The gender also had no effect on CST after subsequent bevacizumab injections (ptime < 0.001, psex = 0.884).

The analysis showed no statistically significant differences for BCVA_relative_ (*p* = 0.954) and CST_relative_ (*p* = 0.645) between the BCVA_≤75_ and the BCVA_>75_ groups (Table 7).

## 4. Discussion

We investigated the effects of bevacizumab treatment (9 injections over 12 months) in patients with diabetic macular edema who were classified as unsatisfactory (BCVA_≤75_ group) and satisfactory (BCVA_>75_ group) responders to the treatment. The cut-off point was chosen deliberately and related to the number of ETDRS letters read by the patients with normal or near-normal visual acuity [11,12,13]. We observed that total contrast leakage area, contrast leakage area in the far periphery and posterior zone after bevacizumab treatment was reduced in each BCVA score group, but the examined parameters were significantly higher in the BCVA_≤75_ group than in the BCVA_>75_ group. The comparative analyzes showed that the macular CST in the BCVA_>75_ group was lower than in the BCVA_≤75_ score group after each subsequent injection.

The obtained results showed that BCVA negatively correlated with baseline macular CST, total MA count, contrast leakage area and retinal non-perfusion area. Bressler et al. showed that based on CST, the changes between baseline and 2 years visual acuity values after 6 injections of anti-VEGF monthly therapy were similar in eyes with and without persistent DME [18]. Protocol T, an anti-VEGF treatment regimen for DME designed by Diabetic Retinopathy Clinical Research Network, showed that 2 years after the treatment, the change in CST after 3 anti-VEGF injections was a weak determinant of a patient’s subsequent visual acuity outcome [19,20]. In our study, we observed that a 100 μm increase in CST corresponded to a decrease by about 5.3 ETDRS letters in patients’ BCVA. We also found that both CST and BCVA correlated with total MA count, contrast leakage area and NPA: macular CST correlated positively, and BCVA correlated negatively with the diabetic retinal abnormalities’ extent. We found that the IVB treatment was more effective in patients at the earlier DME stage, who expressed higher initial BCVA and lower CST. The more ETDRS letters the patient could read at the beginning of bevacizumab therapy, the smaller the change in macular CST was observed. This observation is consistent with other anti-VEGF treatments presented in studies trying to classify DME using ultra-wide-field fluorescein angiography [17,21].

The literature shows that patients with worse baseline visual acuity present more remarkable improvement after anti-VEGF therapy. Ying et al. [19] showed that one year after ranibizumab or bevacizumab treatment for neovascular age-related macular degeneration (AMD), the patients with worse baseline visual acuity (VA) had lower mean VA scores. However, the mean increase in VA and the proportion of ≥3-line improvement were most remarkable for the patients with baseline VA of 20/100 to 20/160 and were lowest for the patients with VA of 20/40 or better. Even though patients with worse baseline visual acuity showed more remarkable improvement before one-year follow-up, the average improvement did not result in the same VA level for all participants at one-year follow-up [22]. Chong and Mitchel’s study [23] on patients with DME after ranibizumab and laser photocoagulation therapy showed that baseline BCVA is a strong predictor of BCVA changes three years after the therapy. Patients with lower VA showed more remarkable improvement than those with better initial vision [23].

Our study showed that patients with strongly impaired visual acuity showed the most remarkable relative change in macular CST and BCVA. Analysis of both studied groups showed that reduction of vascular leakage improves visual acuity. Thus, patients with lower leakage area after therapy achieved better visual acuity. Moreover, patients with low ETDRS baseline scores showed a larger relative increase in BCVA after the therapy, while patients with good ETDRS baseline scores showed smaller relative improvement. Both groups of patients improved by the same number of letters after the treatment, but the patients that ultimately were assigned to the group with the lower final BCVA (reading ≤75 ETDRS letters) eventually read fewer letters than the patients that were assigned to the group with higher final BCVA score (reading >75 ETDRS letters). It strongly suggests that, in clinical trials, the improvement in visual acuity, measured with the number of ETDRS letters, is as crucial as the stability of the improvement after the treatment. However, focusing on improving visual acuity alone can be misleading because patients with the greatest improvement in visual acuity do not necessarily represent the patients with the best visual acuity.

In addition, our results indicate that DR severity is associated with NPA. We showed that excellent improvement in visual acuity (final BCVA > 75 ETDRS letters) could be achieved with the protocol applied in this study, regardless of the retinal non-perfusion and vascular leakage advancement in all retinal zones and DME severity [10]. NPA did not influence the final BVCA results in both study groups, so we can conclude that retinal non-perfusion did not affect the effectiveness of the therapy in both study groups. Analysis of both studied groups showed that reduction of vascular leakage improves visual acuity. Thus, patients with lower leakage area after therapy achieved better visual acuity. Our results agree with Ehlers et al. [24], who used quantitative ultra-wide-field angiography to diagnose the severity of diabetic retinopathy in 339 eyes. They proved that pan-retinal leakage index, pan-retinal ischemic index and pan-retinal microaneurysm count correlate with DR severity [24].

We observed an improvement in visual acuity and decreased CST after the bevacizumab treatment we applied (9 injections over 12 months) in both groups of patients. The studies reporting effects of DME treatment with different types of anti-VEGF showed that approximately 50% of patients treated for six months with anti-VEGF presented a significant CST reduction [25]. Other studies reported that only 20.7% of patients were considered good responders, in terms of CST reduction, to 6-months anti-VEGF therapy [26]. Some studies showed that a subgroup of patients with a lower BCVA at baseline showed better visual acuity and anatomic outcomes with ranibizumab [27,28]. We also observed that NPDR severity and post-treatment BCVA score are related. In the BCVA_≤75_ group, the highest percentage of patients presented moderate NPDR, while in the BCVA_>75_ group, 40% of patients presented moderate NPDR, and 35% of them presented mild NPDR. We noted that patients from the BCVA_>75_ group had better visual acuity and lower CST before the therapy than patients from the BCVA_≤75_ group. We concluded that DME therapy with bevacizumab should not only start when macular CST is high, and the initial visual acuity is poor because then the probability of obtaining a BCVA score of >75 ETDRS letters after the treatment is lower. Bevacizumab therapy gave the same results when comparing the progression of the number of ETDRS letters read by patients from both analyzed groups. The effect of the IVB therapy had a similar effect on the visual acuity improvement: patients from both groups gained a similar number of ETDRS letters read after completing the therapy. However, patients from the BCVA_≤75_ group initially read fewer letters, and therefore, after the therapy, their visual acuity was lower. A longer duration of DME is associated with poorer outcomes, which suggests the need to initiate the therapy as early as possible. The variability in treatment outcomes possibly results from a different anatomical and functional response to anti-VFGF treatment [23]. Bressler et al. [29] suggested that the correlation between lower visual acuity assessed at the beginning of the treatment and higher degree of visual acuity improvement may, at least partially, result from the degree of improvement possible at the time of treatment for those with better visual acuity [29]. Nevertheless, similarly to another work by Bressler et al. [18], we can conclude that the applied therapy is more effective when started early, in DME patients with better baseline visual acuity and lower CST. Since bevacizumab is more cost-effective than other anti-VEGF agents used in DME treatment (ranibizumab or aflibercept) [14,30,31] such an approach is even more feasible. Initially, IVB was used for treating breast, lung and gastrointestinal tract malignancies. However, many trials have already assessed the safety and effectiveness of its use in ophthalmic disease treatment. It is also listed for treating eye disease on the World Health Organization’s List of Essential Medicines [14,30,31,32].

Bressler et al. observed that younger patients presented better vision improvement one year after ranibizumab injections with triamcinolone and laser for center-involved DME [29]. Even though it is unclear why, younger age also correlated with superior vision outcomes in patients treated with ranibizumab for neovascular age-related macular degeneration [33]. However, we found no relation between patients’ age, BMI and type 2 diabetes duration and their BCVA score or CST values in both groups of patients subjected to bevacizumab treatment. Systemic characteristics such as duration of diabetes and glycemic control play a substantial role in diabetic retinopathy development [34]. The integrated data from the VISTA and VIVID studies showed that there is no link between improvement in DRSS (diabetic retinopathy severity score) and baseline age, duration of diabetes, HbA1c, BMI, BCVA and CST [35]. In our study, the factors associated with better visual acuity after nine intravitreal bevacizumab injections, as assessed by the treating ophthalmologist and supported by the results, were as follows: lower DR severity, better visual acuity and lower macular CST at the time of treatment initiation. Even though many factors were analyzed, only those mentioned, which can be considered the most basic, turned out to be important in achieving high visual acuity after the end of the first year of treatment. Due to the progress in ophthalmology, the achievement of a comfortable, close-to-normal vision after the treatment is becoming a frequent expectation among patients. With treatment started early enough, when the vision of the diabetic patient is not severely impaired yet, this expectation can be easily met. In some countries, the reimbursement for anti-VGEF injections is only available to patients with worse vision. Our work shows that it is a wrong approach, as patients with low visual acuity at baseline will obviously improve. However, they will not achieve as good visual acuity as patients with better visual acuity at baseline, who currently do not have access to reimbursement for bevacizumab treatment. The study proved that the ophthalmic screening of diabetic retinopathy patients should also target patients with relatively high visual acuity.

Future studies should investigate the association between capillary non-perfusion and visual function parameters and its relationship to DME progression.

## 5. Conclusions

We found that the effectiveness of bevacizumab treatment in improving visual acuity was observed in most patients and was similar in the patients with higher and lower final visual acuity. However, a prerequisite for normal or near-normal vision is to start bevacizumab treatment when visual acuity is not significantly reduced yet. The study proved that the ophthalmic screening of diabetic retinopathy patients should also target patients with relatively high visual acuity.

## Figures and Tables

**Table 1:** Study design and flow of patients with diabetic macular edema (DME) taking part in the experiment assessing the effectiveness of intravitreal bevacizumab treatment.

Stage of Experiment	Activity	Methods
Recruitment	Initial interview	Chart survey
Diagnostics	Initial examination	Slit lamp examination
Qualification	98 patients/eyes qualified	
Diagnostics—before IVB treatment	DME and DR assessment	UWFFA, SS-OCT, ETDRS chart testing
Monitoring—during IVB treatment	IVB injection (I–V every month, VI–IX every two months)	SS-OCT before each intravitreal injection
Diagnostics—after IVB treatment	DME and DR assessment	UWFFA, SS-OCT, ETDRS chart testing
Initial analysis of the results	Distinguishing BCVA_≤75_ (*n* = 41) and BCVA_>75_ (*n* = 57) groups	
Final analysis of the results	Statistical analyses within groups	

Abbreviations: IVB—bevacizumab, BCVA—best-corrected visual acuity, BCVA_≤75_—patients who scored ≤ 75 ETDRS letters 1 month after completing the treatment, BCVA_>75_—patients who scored > 75 ETDRS letters 1 month after completing the treatment, DME—diabetic macular edema, DR—diabetic retinopathy, ETDRS—Early Treatment Diabetic Retinopathy Study, SS-OCT—swept-source optical coherence tomography, UWWFA—ultra-wide-field fluorescein angiography.

**Table 2:** Diabetic retinopathy qualitative variables of patients with diabetic macular edema (DME) qualified for the study described as unsatisfactory (BCVA_≤75_, *n* = 41) or satisfactory (BCVA_>75_, *n* = 57) responders to the intravitreal bevacizumab treatment. The data were compared using chi-squared (χ^2^) test. Statistical significance was set at *p* < 0.05.

Variable	Variants	BCVA_≤75_ *n* = 41	BVCA_>75_ *n* = 57	χ^2^	*p*
Sex	female	24 (59%)	33 (58%)	0.01	0.949
	male	17 (41%)	24 (42%)		
Lens status	phakic	19 (46%)	31 (54%)	0.618	0.432
	pseudophakic	22 (54%)	26 (46%)		
Eye	right	20 (49%)	26 (46%)	0.10	0.757
	left	21 (51%)	31 (54%)		
DME	focal	17 (41%)	31 (54%)	1.594	0.207
	diffuse	24 (59%)	26 (46%)		
NPA	yes	21 (51%)	28 (49%)	0.04	0.838
	no	20 (49%)	29 (51%)		
Contrast vascular leakage (far periphery)	yes	16 (39%)	18 (32%)	0.58	0.445
	no	25 (61%)	39 (68%)		
Contrast vascular leakage (mid-periphery)	yes	18 (44%)	19 (33%)	1.13	0.287
	no	23 (56%)	38 (67%)		
Contrast vascular leackage (posterior zone)	yes	19 (46%)	34 (60%)	1.70	0.192
	no	22 (54%)	23 (40%)		
NPDR before therapy	mild	6 (15%)	20 (35%)	7.07	< 0.05
	moderate	27 (66%)	23 (40%)		
	severe	8 (19%)	14 (25%)		
Hypertension	yes	29 (71%)	36 (63%)	0.25	0.619
	no	12 (29%)	21 (37%)		
Hypercholesterolemia	yes	25 (61%)	30 (53%)	0.67	0.412
	no	16 (39%)	27 (47%)		
Ischemic heart disease	yes	5 (12%)	12 (21%)	1.30	0.253
	no	36 (88%)	45 (79%)		
Kidney failure	yes	5 (12%)	2 (4%)	2.71	0.100
	no	36 (88%)	55 (96%)		
Insulin treatment	yes	23 (56%)	33 (58%)	0.61	0.434
	no	18 (44%)	24 (42%)		
Anticoagulant treatment	yes	2 (5%)	50 (88%)	0.805	0.370
	no	39 (95%)	7 (12%)		

Abbreviations: BCVA—best-corrected visual acuity, BCVA_≤75_—patients who scored ≤ 75 ETDRS letters 1 month after completing the treatment, BCVA_>75_—patients who scored >75 ETDRS letters 1 month after completing the treatment, DME—diabetic macular edema, NPA—non-perfusion area, NPDR—non-proliferative diabetic retinopathy.

**Table 3:** Correlations of best-corrected visual acuity (BCVA) and macular central subfield thickness (CST) with selected variables in patients (*n* = 98) with diabetic macular edema (DME) before bevacizumab therapy. The results are expressed using r Pearson’s or ρ Spearman’s correlation coefficients. Statistical significance was set at *p* < 0.05.

Examined Parameter	Variables as Measured before Therapy	r/ρ *	*p*
BCVA (ETDRS letters)	CST (μm)	−0.591	<0.001
	Total MA count (*n*)	−0.285	<0.01
	MA count in posterior zone (*n*)	−0.377	<0.001
	Total contrast leakage area (mm^2^)	−0.343 *	<0.001
	Contrast leakage area in far periphery (mm^2^)	−0.252 *	<0.05
	Contrast leakage area in mid-periphery (mm^2^)	−0.315 *	<0.01
	Contrast leakage area in posterior zone (mm^2^)	−0.287 *	<0.01
	Total NPA (mm^2^)	−0.272 *	<0.01
	NPA in far periphery (mm^2^)	−0.303 *	<0.01
	NPA in mid-periphery (mm^2^)	−0.314 *	<0.01
	NPA in posterior zone (mm^2^)	−0.104 *	0.309
	Age (years)	−0.053 *	0.602
	T2DM duration (year)	−0.024	0.818
	BMI (kg/m^2^)	−0.144	0.156
	HbA1c (%)	−0.149	0.142
CST (μm)	BCVA (ETDRS letters)	−0.591	<0.001
	Total MA count (*n*)	0.389	<0.001
	MA count in posteriori (*n*)	0.625	<0.001
	Total contrast leakage area (mm^2^)	0.414 *	<0.001
	Leakage area in far periphery (mm^2^)	0.165 *	0.104
	Contrast leakage area in mid-periphery (mm^2^)	0.130 *	0.203
	Contrast leakage area in posterior zone (mm^2^)	0.515 *	<0.001
	Total NPA (mm^2^)	0.210 *	<0.05
	NPA in far periphery (mm^2^)	0.249 *	<0.05
	NPA in mid-periphery (mm^2^)	0.163 *	0.109
	NPA in posterior zone (mm^2^)	0.033 *	0.748
	Age (years)	0.150	0.141
	T2DM duration (years)	−0.047	0.647
	BMI (kg/m^2^)	0.148	0.147
	HbA1c (%)	0.268	<0.01

Legend: *—ρ Spearman’s correlation coefficient. Abbreviations: BCVA—best-corrected visual acuity, BMI—body mass index, CST—central subfield thickness, DME—diabetic macular edema, ETDRS—Early Treatment Diabetic Retinopathy Study, HbA1c—glycated hemoglobin, MA—microaneurysm, NPA—non-perfusion area, T2DM—type 2 *diabetes mellitus.*

**Table 4:** NPDR (non-proliferative diabetic retinopathy) severity in patients (*n* = 98) with diabetic macular edema (DME) before and after bevacizumab therapy.

Patients with NPDR Symptoms before Therapy [*n*]		Patients with NPDR Symptoms after Therapy [*n*]		
		Mild	Moderate	Severe
Mild	26	26 (27%)	0 (0%)	0 (0%)
Moderate	50	31 (32%)	19 (19%)	0 (0%)
Severe	22	5 (5%)	15 (15%)	2 (2%)

Abbreviations: DME—diabetic macular edema, NPDR—non-proliferative diabetic retinopathy.

**Table 5:** Correlations of non-proliferative diabetic retinopathy (NPDR) with selected variables in patients (*n* = 98) with diabetic macular edema (DME) before bevacizumab therapy. The results are presented as ρ Spearman’s correlation coefficients. Statistical significance was set at *p* < 0.05.

Variables Correlating with NPDR	ρ	*p*
Total contrast leakage area	0.773	<0.001
Contrast leakage area in far periphery	0.545	<0.001
Contrast leakage in mid-periphery	0.658	<0.001
Contrast leakage in posterior zone	0.643	<0.001
Total NPA	0.679	<0.001
NPA in far periphery	0.514	<0.001
NPA in mid-periphery	0.663	<0.001
NPA in posterior zone	0.649	<0.001

Abbreviations: DME—diabetic macular edema, NPA—non-perfusion area, NPDR—non-proliferative diabetic retinopathy.

**Table 6:** Comparison of contrast leakage areas and non-perfusion areas before and after bevacizumab treatment in the BCVA_≤75_ group (*n* = 41) and the BCVA_>75_ group (*n* = 57) of patients with diabetic macular edema. The results are presented as median (lower;upper quartile) Me (Q1;Q3). Statistical significance was set at *p* < 0.05.

Examined Parameters	Study Group	Before Bevacizumab Therapy	After Bevacizumab Therapy	*p*_before vs._ _after_
Total contrast leakage area [mm^2^]	BCVA_≤75_	23.0 (18.0;67.0)	9.0 (4.0;15.0)	<0.001
	BCVA_>75_	29.0 (14.0;58.0)	4.0 (2.0;10.0)	<0.001
	*p*_≤75 vs.__>75_	0.757	<0.05	
Contrast leakage area in far periphery [mm^2^]	BCVA_≤75_	0.0 (0.0;9.0)	0.0 (0.0;0.0)	<0.001
	BCVA_>75_	0.0 (0.0;10.0)	0.0 (0.0;0.0)	<0.001
	*p*_≤75 vs.__>75_	0.743	<0.05	
Contrast leakage area in mid-periphery [mm^2^]	BCVA_≤75_	4.0 (0.0;1.0)	0.0 (0.0;3.0)	<0.001
	BCVA_>75_	3.0 (0.0;14.5)	0.0 (0.0;2.0)	<0.001
	*p*_≤75 vs.__>75_	0.927	0.938	
Contrast leakage area in posterior [mm^2^]	BCVA_≤75_	20.0 (14.0;31.0)	6.0 (4.0;12.0)	<0.001
	BCVA_>75_	20.0 (10.0; 31.0)	4.0 (2.0;7.0)	<0.001
	*p*_≤75 vs.__>75_	0.572	<0.05	
Total NPA [mm^2^]	BCVA_≤75_	11.0 (0.0;29.0)	2.0 (0.0;16.0)	<0.001
	BCVA_>75_	0.0 (0.0;28.0)	0.0 (0.0;4.0)	<0.001
	*p*_≤75 vs.__>75_	0.561	0.115	
NPA in far periphery [mm^2^]	BCVA_≤75_	0.0 (0.0;18.0)	0.0 (0.0;9.0)	<0.01
	BCVA_>75_	0.0 (0.0;4.0)	0.0 (0.0;0.0)	<0.01
	*p*_≤75 vs.__>75_	0.157	0.063	
NPA in mid-periphery [mm^2^]	BCVA_≤75_	0.0 (0.0;8.0)	0.0 (0.0;2.0)	<0.001
	BCVA_>75_	0.0 (0.0;5.0)	0.0 (0.0;0.0)	<0.001
	*p*_≤75 vs.__>75_	0.610	0.367	
NPA in posterior [mm^2^]	BCVA_≤75_	0.0 (0.0;4.0)	0.0 (0.0;2.0)	<0.01
	BCVA_>75_	0.0 (0.0;4.0)	0.0 (0.0;1.0)	<0.001
	*p*_≤75 vs.__>75_	0.804	0.640	

Abbreviations: BCVA—best-corrected visual acuity, BCVA_≤75_—patients who scored ≤75 ETDRS letters 1 month after completing the treatment, BCVA_>75_—patients who scored > 75 ETDRS letters 1 month after completing the treatment, NPA—non-perfusion area.

**Table 7:** Comparison of BCVA_relative_ and CST_relative_ in the BCVA_≤75_ group (*n* = 41) and the BCVA_>75_ group (*n* = 57) of patients with diabetic macular edema. The results are presented as median (upper; lower quartile) Me (Q1;Q3). Statistical significance was set at *p* < 0.05.

Variables	BCVA_≤75_ (*n* = 41)	BCVA_>75_ (*n* = 57)	*p*
BCVA_relative_	13.6 (5.0;19.6)	10.7 (8.0;17.3)	0.954
CST_relative_	−33.4 (−39.4;−12.9)	−27.6 (−39.8;−17.1)	0.645

Abbreviations: BCVA—best-corrected visual acuity, BCVA_≤75_—patients who scored ≤ 75 ETDRS letters 1 month after completing the treatment, BCVA_>75_—patients who scored > 75 ETDRS letters 1 month after completing the treatment, CST—central subfield thickness.

## Data Availability

The data presented in this study are available on request from the corresponding author.

## References

[1] Ehlken C., Ziemssen F., Eter N., Lanzl I., Kaymak H., Lommatzsch A., Schuster A.K. (2020). Systematic review: Non-adherence and non-persistence in intravitreal treatment. Graefe’s Arch. Clin. Exp. Ophthalmol..

[2] Shimizu K., Kobayashi Y., Muraoka K. (1981). Mid-peripheral fundus involvement in diabetic retinopathy. Ophthalmology.

[3] Friberg T.R., Gupta A., Yu J., Huang L., Suner I., Puliafito C.A., Schwartz S.D. (2008). Ultrawide angle fluorescein angiographic imaging: A comparison to conventional digital acquisition systems. Ophthalmic Surg. Lasers Imaging.

[4] Davis M.D., Norton W.D., Myers F.L., Goldberg M.F., Fine S.L. (1968). Airlie classification of diabetic retinopathy. Symposium on the Treatment of Diabetic Retinopathy.

[5] Fan W., Wang K., Falavarjani K.G., Sagong M., Uji A., Ip M., Wykoff C.C., Brown D.M., van Hemert J., Sadda S.V.R. (2017). Distribution of nonperfusion area on ultra-widefield fluorescein angiography in eyes with diabetic macular edema: DAVE Study. Am. J. Ophthalmol..

[6] Aiello L.P., Odia I., Glassman A.R., Melia M., Jampol L.M., Bressler M.N., Kiss S., Silva P.S., Wykoff C.C., Sun J.K. (2019). Comparison of Early Treatment Diabetic Retinopathy Study standard 7-field imaging with ultrawide-field imaging for determining severity of diabetic retinopathy. JAMA Ophthalmol..

[7] Wylęgała A., Bolek B., Wylęgała E. (2020). Trends in optical coherence tomography angiography use in university clinic and private practice setting between 2014–2018. Expert Rev. Med. Devices.

[8] Lee R., Wong T.Y., Sabanayagam C. (2015). Epidemiology of diabetic retinopathy, diabetic macular edema and related vision loss. Eye Vis..

[9] Ogurtsova K., da Rocha Fernandes J.D., Huang Y., Linnenkamp U., Guariguata L., Cho N.H., Cavan D., Shaw J.E., Makaroff L.E. (2017). IDF Diabetes Atlas: Global estimates for the prevalence of diabetes for 2015 and 2040. Diabetes Res. Clin. Pract..

[10] Sędziak-Marcinek B., Teper S., Chełmecka E., Wylęgała A., Bias M., Wylęgała E. (2021). Diabetic macular edema treatment with bevacizumab does not depend on the retinal non-perfusion presence. J. Diabetes Res..

[11] Baker C.W., Glassman A.R., Beaulieu W.T., Antoszyk A.N., Browning D.J., Chalam K.V., Gover S., Jampol L.M., Jhaveri C.D., Melia M. (2019). Effect of Initial management with aflibercept vs laser photocoagulation vs observation on vision loss among patients with diabetic macular edema involving the center of the macula and good visual acuity. JAMA.

[12] Glassman A.R., Baker C.W., Beaulieu W.T., Bressler N.M., Punjabi O.S., Stockdale C.R., Wykoff C.C., Jampol L.M., Sun J.K., DRCR Retina Network (2020). Assessment of the DRCR Retina Network approach to management with initial observation for eyes with center-involved diabetic macular edema and good visual acuity: A secondary analysis of a randomized clinical trial. JAMA Ophthalmol..

[13] Zafar S., Smith K., Boland M.V., Weng C.Y., Solomon S., Channa R. (2020). Real-world outcomes among eyes with center-involving diabetic macular edema and good visual acuity. Curr. Eye Res..

[14] Wells J.A., Glassman A.R., Ayala A.R., Jampol L.M., Aiello L.P., Antoszyk A.N., Arnold-Bush B., Baker C.W., Bressler N.M., Diabetic Retinopathy Clinical Research Network (2015). Aflibercept, bevacizumab, or ranibizumab for diabetic macular edema. N. Eng. J. Med..

[15] Michaelides M., Kaines A., Hamilton R.D., Fraser-Bell S., Rajendram R., Quhill F., Boos C.J., Xing W., Egan C., Peto T. (2010). A prospective randomized trial of intravitreal bevacizumab or laser therapy in the management of diabetic macular edema (BOLT Study) 12-month data: Report 2. Ophthalmology.

[16] Cai S., Bressler N.M. (2017). Aflibercept, bevacizumab or ranibizumab for diabetic macular oedema: Recent clinically relevant findings from DRCR.net protocol T. Curr. Opin. Ophthalmol..

[17] Fang M., Fan W., Shi Y., Ip M.S., Wykoff C.C., Wang K., Falavarjani K.G., Brown D.M., van Hemert J., Sadda S.R. (2019). Classification of regions of nonperfusion on ultra-widefield fluorescein angiography in patients with diabetic macular edema. Am. J. Ophthalmol..

[18] Bressler S.B., Odia I., Maguire M.G., Dhoot D.S., Glassman A.R., Jampol L.M., Marcus D.M., Solomon S.D., Sun J.K., Diabetic Retinopathy Clinical Research Network (2019). Factors associated with visual acuity and central subfield thickness changes when treating diabetic macular edema with anti-vascular endothelial growth factor therapy: An exploratory analysis of the protocol T randomized clinical trial. JAMA Ophthalmol..

[19] Bressler N.M., Beaulieu W.T., Maguire M.G., Glassman A.R., Blinder K.J., Bressler S.B., Gonzalez V.H., Jampol L.M., Melia M., Sun J.K. (2018). Early response to anti-vascular endothelial growth factor and two-year outcomes among eyes with diabetic macular edema in protocol T. Am. J. Ophthalmol..

[20] Bressler N.M., Beaulieu W.T., Glassman A.R., Blinder K.J., Bressler S.B., Jampol L.M., Melia M., Wells J.A., Diabetic Retinopathy Clinical Research Network (2018). Persistent macular thickening following intravitreous aflibercept, bevacizumab, or ranibizumab for central-involved diabetic macular edema with vision impairment: A secondary analysis of a randomized clinical trial. JAMA Ophthalmol..

[21] Xue K., Yang E., Chong N.V. (2017). Classification of diabetic macular oedema using ultra-widefield angiography and implications for response to anti-VEGF therapy. Br. J. Ophthalmol..

[22] Ying G.-S., Huang J., Maguire M.G., Jaffe G.J., Grunwald J.E., Toth C., Daniel E., Klein M., Pieramici D., Wells J. (2013). Baseline predictors for one-year visual outcomes with ranibizumab or bevacizumab for neovascular age-related macular degeneration. Ophthalmology.

[23] Chong V., Mitchell P. (2013). Baseline predictors of 3-year responses to ranibizumab and laser photocoagulation therapy in patients with visual impairment due to diabetic macular edema (DME). Eur. J. Ophthalmol..

[24] Ehlers J.P., Jiang A.C., Boss J.D., Hu M., Figueiredo N., Babiuch A., Talcott K., Sharma S., Hach J., Le T. (2019). Quantitative ultra-widefield angiography and diabetic retinopathy severity: An assessment of panretinal leakage index, ischemic index and microaneurysm count. Ophthalmology.

[25] Rodrigues M.W., Cardillo J.A., Messias A., Siquiera R.C., Scott I.U., Jorge R. (2020). Bevacizumab versus triamcinolone for persistent diabetic macular edema: A randomized clinical trial. Graefe’s Arch. Clin. Exp. Ophthalmol..

[26] Elman M.J., Qin H., Aiello L.P., Beck R.W., Bressler N.M., Ferris F.L., Glassman A.R., Maturi R.K., Melia M., Diabetic Retinopathy Clinical Research Network (2012). Intravitreal ranibizumab for diabetic macular edema with prompt versus deferred laser treatment: Three-year randomized trial results. Ophthalmology.

[27] Aiello L.P., Beck R.W., Bressler N.M., Browning D.J., Davis M., Ferris F.L., Glassman A.R., Maturi R.K., Diabetic Retinopathy Clinical Research Network, Writing Committee (2011). Rationale for the diabetic retinopathy clinical research network protocol for center-involved diabetic macular edema. Ophthalmology.

[28] Vader M.J.C., Schauwvlieghe A.-S.M.E., Verbraak F.D., Dijkman G., Hooymans J.M.M., Los L.I., Zwinderman A.H., Peto T., Hoyng C.B., van Leeuven R. (2020). Comparing the efficacy of bevacizumab and ranibizumab in patients with diabetic macular edema (BRDME). Ophthalmol. Retin..

[29] Bressler S.B., Qin H., Beck R.W., Chalam K.V., Kim J.E., Melia M., Wells J.A., Diabetic Retinopathy Clinical Research Network (2012). Factors associated with changes in visual acuity and central subfield thickness at 1 year after treatment for diabetic macular edema with ranibizumab. Arch. Ophthalmol..

[30] Bro T., Derebecka M., Jørstad Ø.K., Grzybowski A. (2020). Off-label use of bevacizumab for wet age-related macular degeneration in Europe. Graefe’s Arch. Clin. Exp. Ophthalmol..

[31] Stefanini F.R., Arevalo J.F., Maia M. (2013). Bevacizumab for the management of diabetic macular edema. World J. Diabetes.

[32] (2019). World Health Organization Model List of Essential Medicines: 21st List 2019.

[33] Boyer D.S., Antoszyk A.N., Awh C.C., Bhisitkul R.B., Shapiro H., Acharya N.R., MARINA Study Group (2007). Subgroup analysis of the MARINA study of ranibizumab in neovascular age-related macular degeneration. Ophthalmology.

[34] White N.H., Sun W., Cleary P.A., Danis R.P., Davis M.D., Hainsworth D.P., Hubbard L.D., LAchin J.M., Nathan D.M. (2008). Prolonged effect of intensive therapy on the risk of retinopathy complications in patients with type 1 *diabetes mellitus*: 10 years after the Diabetes Control and Complications Trial. Arch. Ophthalmol..

[35] Dhoot D.S., Baker K., Saroj N., Vitti R., Berliner A.J., Metzig C., Thompson D., Singh R.P. (2018). Baseline factors affecting changes in diabetic retinopathy severity scale score after intravitreal aflibercept or laser for diabetic macular edema: Post hoc analyses from VISTA and VIVID. Ophthalmology.

